# International investment liberalization, transnational corporations and NCD prevention policy non-decisions: a realist review on the political economy of tobacco, alcohol and ultra-processed food

**DOI:** 10.1186/s12992-021-00784-3

**Published:** 2021-11-24

**Authors:** Penelope Milsom, Richard Smith, Phillip Baker, Helen Walls

**Affiliations:** 1 Department of Global Health and Development, Faculty of Public Health and Policy, London School of Hygiene and Tropical Medicine, 15-17 Tavistock Place, Kings Cross, London, WC1H 9SH UK; 2 College of Medicine and Health, University of Exeter, Exeter, UK; 3 Institute for Physical Activity and Nutrition, School of Exercise and Nutrition Sciences, Deakin University, Geelong, VIC Australia

**Keywords:** Investment liberalization, Foreign investment, Non-communicable diseases, Regulatory chill, Political economy of health

## Abstract

**Background:**

Public health concerns relating to international investment liberalization have centred on the potential for investor-state dispute settlement (ISDS)-related regulatory chill. However, the broader political and economic dimensions that shape the relationship between the international investment regime and non-communicable disease (NCD) policy development have been less well explored. This review aimed to synthesise the available evidence using a political economy approach, to understand why, how and under what conditions transnational corporations may use the international investment regime to promote NCD prevention policy non-decisions.

**Main body:**

**Methods:** Mechanisms explaining why/how the international investment regime may be used by transnational health-harmful commodity corporations (THCCs) to encourage NCD prevention policy non-decisions, including regulatory chill, were iteratively developed. Six databases and relevant grey literature was searched, and evidence was extracted, synthesized and mapped against the various proposed explanatory mechanisms.

**Findings:** Eighty-nine sources were included. THCCs may be incentivised to use the ISDS mechanism since the costs may be outweighed by the benefits of even just delaying regulatory adoption, particularly since the chilling effect tends to ripple out across jurisdictions. Drivers of regulatory chill may include ambiguity in treaty terms, inconsistency in arbitral rulings, potential arbitrator bias and the high cost of arbitration. Evidence indicates ISDS can delay policy adoption both within the country directly involved but also in other jurisdictions. Additionally, governments are adopting standard assessments of public health regulatory proposals for trade and ISDS risk. Various economic, political and industry-related factors likely interact to increase (or decrease) the ultimate risk of regulatory chill. Some evidence indicates that THCCs take advantage of governments’ prioritization of foreign investment over NCD prevention objectives to influence the NCD prevention regulatory environment.

**Conclusions:**

While ISDS-related regulatory chill is a real risk under certain conditions, international investment-related NCD prevention policy non-decisions driven by broader political economy dynamics may well be more widespread and impactful on NCD regulatory environments. There is therefore a clear need to expand the research agenda on investment liberalization and NCD policy beyond regulatory chill and engage with theories and approaches from international relations and political science, including political economy and power analyses.

**Supplementary Information:**

The online version contains supplementary material available at 10.1186/s12992-021-00784-3.

## Background

It is well known that tobacco, alcohol and unhealthy diets are key risk factors for non-communicable diseases (NCDs) which now account for more than 70% of global deaths annually. Over 85% of preventable NCD deaths occur in low- and middle-income countries (LMICs) [1]. However, as markets for harmful products saturate in high-income countries (HICs), investment into the alcohol, ultra-processed food (UPF) and, in some cases, tobacco sector is increasing in many LMICs, particularly in Asia [2, 3]. This investment allows corporations engaged in the production, distribution and sale of UPF, alcohol and tobacco to reduce production costs, gain efficiencies in distribution, and sell their products at a low cost domestically [4]. Foreign direct investment (FDI) has consequently been associated with increased consumption of health-harmful products in a number of LMICs [5–9]. Public health measures to reduce consumption of these products are in direct tension with the financial objectives of the corporations producing them. As such, these corporations (referred to in this work as transnational health harmful commodity corporations or THCCs) may be increasingly interested in maintaining a limited regulatory environment in these countries. The relevance of understanding the linkages between the liberalization of cross-border capital flows and FDI, growth in the size and transnational reach of multinational corporations, and NCD prevention policy *non*-decisions is therefore increasingly pertinent. Non-decisions are defined in this work as a voluntary decision not to act; an involuntary failure to act; or inaction due to a psychological boundary issue [10].

Concern from the public health community regarding international investment liberalization has largely focused on the ISDS mechanism found in more than 3500 international investment agreements (IIAs) and in over 60 trade agreements including regional and more recently negotiated large multi-lateral trade agreements [11]. The ISDS mechanism provides a pathway for foreign corporations to bypass domestic courts and bring claims for financial compensation against states in private international tribunals when a corporation perceives state action has compromised their investment [12, 13]. ISDS originates from efforts by former colonial powers and international organizations, particularly the World Bank, to maintain influence within newly independent and developing countries [14] and multinational corporations have widely lobbied for its inclusion in IIAs and trade agreements. Public health concerns have centred around the potential for ISDS to be used by THCCs to block new policies aimed at protecting public health or to generate ‘regulatory chill’, a specific type of policy non-decision where a government fails to regulate in the public interest in a timely and effective manner due to a high perceived threat of investment arbitration [12, 15–18]. As such, IIAs potentially provide THCCs with veto power over domestic public health policy decisions [12]. Despite significant recent debates and steps by some countries to reduce their exposure to ISDS, the mechanism remains a standard model for resolving international investment disputes [19].

Public health concern relating to ISDS has not been unwarranted. In 2010 Phillip Morris International filed a dispute against Uruguay (under an agreement with Switzerland) for their tobacco graphic warning labelling regulations. The following year Philip Morris Asia initiated a dispute against Australia (under an IIA with Hong Kong) for their proposed standardized tobacco packaging [20, 21]. While the food and alcohol industry have not yet utilized the ISDS mechanism, there is evidence that they are increasingly adopting tobacco industry strategies to influence policymaking [22]. Notably, LMICs may be particularly vulnerable to an investment-related chilling effect on progressive public health policy. Reasons for this potentially include their exposure through IIAs with HICs where the majority of THCCs are domiciled, increasing investment by THCCs in LMICs and the limited administrative, legal technical and financial resources held by LMICs to successfully navigate an investor-state dispute.

A body of literature analysing the potential regulatory chilling effect of IIA obligations on health policy decisions is developing. A 2018 critical review by Schram et al. including 33 articles, outlined the methodological approaches used to study investment dispute-related regulatory chill, described the existing state of knowledge on the issue and developed a conceptual framework of the internalization of IIAs in public policy [23]. However, the broader political and economic dimensions that shape the relationship between the international investment regime and NCD policy development have been less well explored. This work argues that adopting a political economy approach may provide further nuance to understanding how, why and under what circumstances the international investment regime may facilitate certain actors to advance their interests within NCD policy decision-making not only instrumentally (e.g., via threats of investor-state disputes) but also structurally (e.g., by appealing to governments’ interest in attracting foreign investment).

The aim of this realist review therefore, is to synthesise the available evidence to understand why, how and under what conditions international investment liberalization may facilitate THCC influence over NCD prevention policy and to identify potential recommendations for minimizing such influence. While evidence is included from countries across all income groups, the focus, where possible, is on LMICs since they have become the focus for expansion by many THCCs [24–27] but may have limited capacity – financial, institutional, technical and/or strategic – to resist attempts by THCCs’ to influence health policy processes [28].

## Main text

### Methods

The realist review is one of the few mixed review methods that offers a "systematic integration of contextual analysis in order to better understand *how* interventions produce outcomes" [29]. This approach was therefore considered useful for providing insight into not only if international investment liberalization has affected NCD prevention policy action, but also how, why and under what circumstances.

The review was conducted according to a protocol broadly based on Pawson’s five iterative stages: identifying and articulating the explanatory theories; searching for and appraising the evidence; extracting the data; synthesizing the evidence; and drawing conclusions [30]. The reporting of this review adheres to RAMSES publication standards [31].

#### Initial scope of the literature and explanatory theory development

An initial rapid scoping review of relevant international investment and health policy literature was conducted using concept searches, e.g. ‘regulatory/policy chill’ and ‘policy space’ in Scopus and Google Scholar. Citation tracking and snowballing was subsequently also used and grey literature was searched. Relevant explanatory information from different sources was interpreted and synthesized in an iterative process of preliminary theory development.

#### Searching and appraising the evidence

##### Main search

A systematic search of the literature was conducted to identify the most relevant evidence to either support or dispute the initial set of explanatory theories. The final search strategy used a combination of search and indexed terms for the concepts of international trade and investment liberalization, regulatory chill, policy process, relevant transnational corporations and three public health policy areas: tobacco control, alcohol regulation and nutrition (Supplementary Text I) [10]. These concepts were developed iteratively by repeated testing and reviewing of search results in MEDLINE, development/refinement of explanatory theories and subsequent further concept development [10]. The search terms were then developed through repeated testing in six databases: MEDLINE, Global Health, Econlit, SCOPUS, Web of Science and PubMed.

Database searches were undertaken in January 2020 and limited to English language publications from 1st January 2008 to 15th January 2020. It was judged reasonable to limit the search from 2008 onwards given the only more recent interest in international investment treaties by public health researchers. Citation tracking and bibliography searching was conducted on studies of particular relevance to theory development [10].

A search for relevant grey literature was also conducted in Google and Google Scholar and online repositories of the World health Organization, World Trade Organization (WTO), United Nations Conference on Trade and Development (UNCTAD) and International Institute of Sustainable Development [10]. All articles were downloaded to an *Endnote* database and duplicates removed.

##### Inclusion criteria

Inclusion criteria were consistent with Pawson’s approach that the decision be based on the article’s relevance to program theories and explanatory potential; whether it contains discernible ‘nuggets’ of evidence; and evidence of trustworthiness with no article excluded based on a single aspect of quality [32]. The criteria applied are outlined in Table 1.

**Table 1:** Inclusion criteria

**Include the article if**	
• It contains ‘nuggets’ of evidence that provide insight into the review questions, such that even where the aims of the study diverge from the main focus of this review, if a ‘nugget’ of evidence relevant to the review questions is provided, this article is included. AND	
• It is assessed to go beyond a superficial description or commentary, i.e. is a competent attempt at research, enquiry, investigation or study ^58^. This can include qualitative studies using key informant interviews and policy document reviews, surveys, expert legal analyses, case studies, reviews of primary research (if the method was stated) or descriptive models/frameworks (if based on primary data).	
**Exclude the article if**	
• The focus is on agricultural policy, food safety, genetically modified foods/GM food labelling, or biotechnology.	
• It analyses trade and/or investment agreements or an ISDS/WTO dispute but does not also explicitly analyze the impacts (or potential impacts) on health policy processes (prospectively or retrospectively) OR on policy space	
• It examines how trade and/or investment liberalization impacted on health determinants and outcomes but not on health policy processes.	
• Books and book chapters.	

##### Selection and appraisal of documents

Electronic searches yielded 1585 results. A further 55 sources were identified through citation tracking, bibliography and grey literature searches. After removing duplicates, 995 unique sources remained. Following the realist approach and due to the limited literature, an intentionally inclusive approach was taken throughout the selection process [10].

Articles underwent a preliminary screening of their titles and abstracts using the inclusion criteria (Table 1). Commentaries, editorials, opinion pieces, conference abstracts, and data-free models/frameworks were excluded (unless based on empirical evidence or providing key anecdotal evidence) [10]. After a scoping of included literature, this review was narrowed to include just the impact of international investment treaties on the three policy areas (international trade is explored separately [10]). The first reviewer’s screen subsequently resulted in 138 texts being retained for full-text review.

**Fig. 1:**
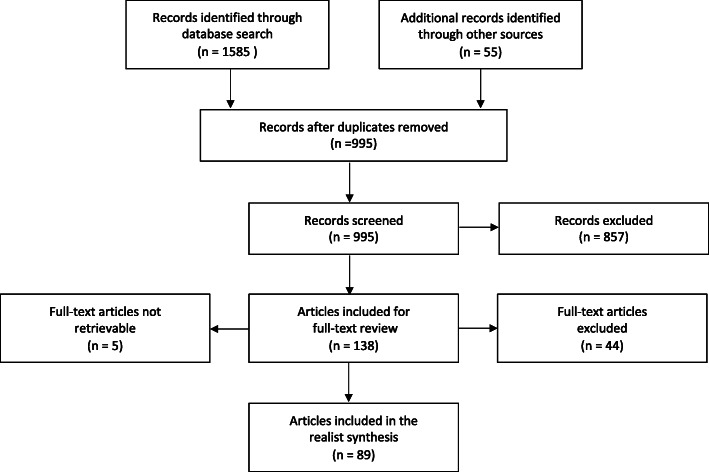
Screening flow diagram

Full texts were retrieved for 133 of the 138 articles. Five articles were not retrievable. The 133 full texts were again assessed for relevance based on the test for inclusion. Full-text review resulted in exclusion of a further 44 articles giving 89 relevant articles (Fig. 1).

Ten percent of the articles were reviewed by the second reviewer both at the preliminary screening and full text review stages. There was 100% inter-reviewer agreement on evidential relevance and study quality at both stages and the remaining texts were assessed for inclusion by the first reviewer only.

Information on study characteristics (e.g. study type, methodological approach, health issues covered) and the rationale for final inclusion/exclusion in the realist synthesis was documented on a screening tool (Supplementary Text II) adapted from a similar set of constructs [33]. It was not possible to apply a single recognized quality appraisal assessment tool to all included articles due to the diversity of disciplines and methods used. Instead, following the realist approach, the focus was on assessing each nugget of relevant evidence identified within an article for its reliability and relevance to theory development [10].

#### Data extraction, analysis and synthesis processes

The final 89 articles included in the synthesis were imported into NVivo (QSR International). ‘Nodes’ were generated for each preliminary explanatory theory. Data considered useful for theory development, including data that supported or challenged preliminary theories and relevant contextual factors, were extracted by the first reviewer. New nodes were generated as additional useful theories emerged and relevant data extracted. The data extracted under each node were imported into a Word document for analysis and synthesis.

## Results

The 89 articles selected for inclusion in the synthesis spanned different disciplines including public health, international law and political science. Articles varied in design and quality and included analyses of investment protection chapters and provisions; analyses of investor-state disputes; surveys and key informant interviews investigating policy-makers’ consideration of investment protection obligations in policy decision-making processes; case-studies of potential regulatory chill responses; and critical analyses of industry and policy documents.

We identified a very limited number of empirical analyses. A major reason for this is likely to be the significant challenges associated with studying policy non-decisions. In addition, our enquiry was inherently multi-disciplinary in nature with legal, political and social science research providing valuable insights. For these reasons analyses based on expert opinion and deductive reasoning as well as empirical studies were included. Notably, analysis of contextual factors was not included as a primary research objective in any of the studies and contextual factors were typically only discussed superficially.

### Political and economic drivers of international investment-related NCD prevention policy non-decisions

Very limited empirical research was identified that primarily or explicitly explored the more political and economic dimensions of how international investment liberalization may shape actor interests and priorities in ways that affect NCD policy.

The neoliberal paradigm is based on the premise that free, open and competitive markets will achieve economic growth, development and shared prosperity [34]. Privatization and liberalization of cross-border capital flows are key elements of neoliberal policy reform [35]. While the relationship between neoliberal reforms and FDI into developing countries is complex, private foreign investment is often, although not always, considered by governments to be a fundamental source of employment, production (and specifically for food, more efficient and reliable supply chains [4]), technology transfer, tax revenue [4, 13] and economic growth. As such, attracting FDI has been identified as a key pillar of the economic development plan in many LMICs, including investment into the processed food, alcohol and, in some cases, tobacco sectors [4]. In their 2018 study, Thow and colleagues found that a dominant ‘Economic Growth’ policy coalition in South Africa held the core belief that employment and economic growth were the primary mechanisms to achieve food and nutrition security [36]. This coalition’s neoliberally-aligned perspective was found to dominate in South Africa’s policy documents governing the food supply. For example, the authors point out that the National Development Plan 2030 includes objectives to increase investment in the agro-processing sector (of which food is a major sub-division) as a means of increasing the production of value-added processed goods and employment, despite national public health goals of reducing obesity and diet-related NCDs [36].

Given many governments’ belief that FDI from private corporations is critical to job creation and economic development, there is considerable scope for THCC’s access to, and influence within, political decision-making spaces to significantly expand [4, 9]. Governments may also be independently willing to refrain from regulating in relation to THCC activities and products in order to attract FDI. Case study evidence supporting this theory was identified in several analyses. In 1995 when Uzbekistan’s domestic tobacco company was privatized in a deal with British American Tobacco (BAT), the company delayed completing its investment (the largest single source of FDI into the country) until proposed regulations (including a ban on tobacco advertising and smoking in public places and health warnings on packages) were replaced with BAT’s voluntary advertising code and cigarette excise tax rates were reduced by 50% [9, 37, 38]. BAT was also identified as having attempted to influence cigarette tax policy in various countries it sought to invest in with documented efforts in Hungary, Belarus, Ukraine, Kyrgyzstan and Cambodia and other TNCs secured similar concessions as conditions of investment [9, 39]. Sarnitsart (2008) reports on documents explaining that when the Laotian Government sold its domestic tobacco monopoly to a foreign investor, a stabilisation clause in the contract committed the government to freezing excise tax on tobacco products for 25 years [40].

In their analysis of Nigerian tobacco control policy, Oladepo and colleagues (2018) report that in 2001 the Nigerian government signed a memorandum of understanding with BAT Nigeria to make an investment of 150 million USD to build a state-of-the-art factory, improve the quality and quantity of locally grown tobacco and develop potential for regional export [41]. Following this there was a 10-year gap between Nigeria’s ratification of the Framework Convention on Tobacco Control (FCTC) in 2005 and the formulation and passage into law of a comprehensive tobacco bill in 2015 [41]. In a 2018 study by Lencucha and colleagues, Malawi tobacco control advocates reported that Japan Tobacco International, which has significant investment in manufacturing in the country, was driving Malawi’s tobacco policy agenda [42].

One study was identified indicating that threat of foreign investor flight may also promote NCD prevention policy non-decisions. Mialon et al. (2016) found that when the Fijian government suggested regulating the food supply to create a healthier food environment, food companies threatened to move their investments out of Fiji and claimed that jobs would be lost. This was reported to result in the government deciding not to pursue such policy options [43].

For THCCs, FDI, as opposed to trade, is considered the preferred and primary method for entering new markets since it can be more cost-effective than exporting products, it avoids trade barriers, optimises the effectiveness of branding and promotional activities and can rapidly assist them to gain market dominance [5, 44]. For example, one study included in the analysis indicated that investment liberalization through NAFTA resulted in huge accelerations of FDI from American processed food corporations into Mexico and, various transnational food corporations have used FDI to expand into LMICs globally [44]. Other literature included descriptions of how transnational tobacco companies (TTCs) have used FDI to gain access into LMICs markets by way of joint ventures or leaf development agreements with state-owned/local companies and the establishment of manufacturing facilities, including in Malawi, Nigeria, Mozambique, Uganda, Zimbabwe and South Africa [37, 45]. In their 2008 case study of Vietnam, Lee and colleagues document how BAT took advantage of the government’s "need" for FDI to negotiate an advantageous joint venture partnership [46].

Once dependent on foreign capital, TTCs have acquired local companies to take control of the market in a number of developing countries, including Cambodia and Vietnam and various Former Soviet countries [37]. During the 1990s TTCs made similar acquisitions of state-owned tobacco companies in Sub-Saharan Africa including RJ Reynolds in Tanzania, and Japan Tobacco International in both Malawi then later in 2011 in Sudan [45]. Transnational alcohol companies have undertaken similar takeovers. For example through a number of share acquisition deals, Diageo became the majority shareholder in India’s United Spirits Limited, taking a leadership position in the Indian alcohol market [47]. Diageo and SABMilller, two of the world’s largest alcohol corporations, have made significant strategic investments in production hubs across LMICs [48]. THCCs also tend to invest at multiple points in a product’s supply chain. While supply chains are often global, they can also exist largely in just one country, for example Coca Cola has invested in cane sugar refinement, beverage production, bottling and refrigeration of sugar-sweetened beverages in Brazil alone [4]. The cross-border investment practices of THCCs described, are likely to have contributed to the accumulation of vast material resources by THCCs. We suggest this in turn translates into material power that THCCs can use to influence regulatory environments in their host countries.

### Drivers of ISDS-related NCD prevention regulatory chill

The majority of literature on international investment and health policy identified in this review focused on ISDS-related regulatory chill. The threat of an investor-state dispute can be used by THCCs as a mechanism to potentially prevent or stall NCD prevention regulatory development at a relatively low cost [12, 49]. Hawkins and Holden (2016) argue that this strategy of influence becomes particularly relevant when THCC legitimacy declines and their access to policymaking spaces is limited, prompting them to claim alternative spaces of influence [12]. Where investor-state disputes are in fact brought, THCCs effectively shift health policy decision-making to private international arbitrators residing within international investment legal venues, where THCCs interests may be prioritised [50, 51]. THCCs have strongly supported both the proliferation of IIAs and more recently have lobbied for the inclusion of investment chapters, and specifically ISDS, in multilateral trade agreements. For example, before the US withdrew from the agreement, Phillip Morris International submitted comments to the US Trade Representative indicating its support for protection of investor rights in the Transpacific Partnership Agreement (TPPA), describing the ISDS provision as "vital" [4, 13, 49].

#### Incentives for THCCs to use ISDS

In their 2013 analysis, Ty points out that there are minimal restrictions on the initiation of an investment dispute under the majority of IIAs giving THCCs wide discretion to file claims [52]. Others suggest that pursuing an investor-state challenge, regardless of ultimate success, may be in the corporate interest, since the economic value of simply delaying policy implementation via lengthy legal processes may outweigh the expense associated with the arbitration itself [13, 53]. This is supported by evidence that despite repeated legal advice that the TRIPS agreement provided no protection against tobacco health warning labelling or plain packaging, tobacco companies continued to threaten that expropriation of the companies’ intellectual property would result in significant financial damages. Crosbie and Glantz (2014) argue this “delayed the progress on large graphic health warning labels and plain packaging for over a decade” [54]. When plain packaging was again on the agenda in 2011, Phillip Morris continued to use the same unsupported TRIPS-based argument and eventually initiated an investor-state dispute on these grounds [49]. Australia and Uruguay’s tobacco control-related ISDS cases lasted for four and 6 years, respectively [17], causing significant delays in policy adoption.

Further incentive for THCCs to proceed to an investor-state dispute comes from evidence of its ripple effect across jurisdictions. A number of analyses pointed out that by strategically targeting countries attempting to introduce precedential public health policy, THCCs may prevent a so-called domino effect regionally and globally [12, 51, 53]. Gruszczynski (2014) for example argues that the key motivation for transnational tobacco corporations’ particularly aggressive opposition to plain packaging in Australia, including through an investor-state dispute, “is the precedential nature of the new law and a fear that the packaging requirements will be copied in other jurisdictions”. Gruszczynski goes on to state that, given Australia’s strong legal position, the tobacco industry’s “approach sends a strong signal to other countries contemplating the introduction of plain packaging laws that any such initiative may be challenged. This in turn can have a chilling effect on national regulatory initiatives of other states” [53]. This argument is supported by reports that a number of countries delayed adoption of standardized packaging during this time [17, 53]. Conversely, THCCs may exercise a degree of caution in proceeding to investment arbitration since it carries the risk that their arguments will be dismissed as illegitimate, undermining future use of such arguments in lobbying activities [49].

#### Ambiguity of international investment agreement rules

A number of analyses also identified that in threatening or pursing an investor-state dispute, corporations take advantage of ambiguity in the definition of key terms/obligations within IIAs. Such ambiguity effectively broadens the scope of investment protection and therefore the number of opportunities for THCCs to challenge regulations, including those related to harmful products [13, 52, 55–61]. ‘Investment’ itself is also defined very broadly in most investment treaties [17]. In NAFTA (replaced in 2020 by the United States-Mexico-Canada Agreement), and also in more recent agreements including the Comprehensive and Progressive Agreement for Transpacific Partnership (CPTPP) signed between 11 Pacific rim countries (after the US withdrew from the TPPA), investment is defined as applying to any assets characterised by "a commitment of capital or other resources, expectation of gain or profit, or assumption of risk" [62–64]. Mitchell and Wurzberger (2011) argue that this makes it very difficult for a country to avoid investment arbitration on the basis that a THCC bringing a claim does not have a relevant investment [62]. The CPTPP’s broad definition could potentially make trademarks, and therefore packaging and labelling of risk products, a covered investment protected under IIAs [65]. The meaning of ‘indirect expropriation’ is also highly debated [17], the details of which are further explored in Supplementary Text III which provides a review of the literature on IIAs investment protection obligations relevant to risk commodity regulatory space.

Additionally, Cooper and colleagues (2014) point out that arbitration panels are required to interpret IIA rules based on the agreement’s overall purpose and objectives outlined in the preamble [66]. Unless health protection provisions are included in the preamble or within the body of the agreement, Hawkins and Holden (2016) further highlight that arbitrators have “no obligation to balance trade and investment liberalisation against other competing social goods” [12]. In disputes brought under NAFTA for example, arbitrators were required to interpret investor rights under the Investment Chapter in the context of NAFTA’s overall objectives which were entirely commercial, although the preamble did include resolutions to preserve states’ flexibility to safeguard public welfare [66]. While NAFTA also included a provision within Article 1101 that appeared to carve out public health measures from liability under the investment protection chapter, these measures were required to be “otherwise consistent” with the chapter [66]. Cooper and colleagues argue that as a result, Article 1101 had “not effectively shielded many public interest measures, nor deterred investors from bringing claims” [66].

Overall, the vagueness of substantive rules and broad protection offered to corporations in IIAs may contribute further to the uncertainty for governments in assessing a measure’s potential to trigger an investor-state dispute. A number of sources identified that this may encourage governments to take a risk-averse approach and pursue a weakened regulation or refrain from regulating entirely, including for measures that would in fact be compliant with international investment law [16, 55, 59, 67]. In their analysis of ISDS and regulatory chill, Tienhaara broadly stated that “substantial ambiguity exists in the text of the TPP(A). Ambiguity equals regulatory uncertainty and thus regulatory chill remains a concern.” [68].

#### Inconsistency in interpretation of international investment rules

A number of legal analyses found investment law interpretation and arbitral outcomes in cases with relevance to public health have been somewhat unpredictable and inconsistent, and point out that this makes it challenging for governments to assess their risk of attracting or losing an investor-state dispute [57, 58, 69]. A 2013 UNCTAD report observed “divergent legal interpretations of identical or similar treaty provisions and differences in the assessment of the merits of cases involving the same facts” [70]. Two examples identified that reflect inconsistency in ISDS rulings include Chemtura Corp Vs Canada and Tecmed Vs Mexico. In 1998 Chemtura Corp filed an investor-state dispute  against Canada arguing NAFTA violations regarding a ban on the use of lindane, a hazardous pesticide [71]. The arbitration panel rejected Chemtura’s case stating that "[T] he rule of Chapter 11 Tribunal is not to second-guess the correctness of the science-based decision-making of highly specialized regulatory agencies" [71]. In contrast, Tecmed filed an ISDS claim against Mexico in 2003 for revoking their permit to operate a landfill due to violation of various health and environmental regulations. In this case the arbitration panel stated "we find no principle stating that regulatory administrative actions are per se excluded from the scope of the Agreement, even if they are beneficial to society as a whole —such as environmental protection" [67].

Various analyses identified a number of different factors that likely contribute to the inconsistency in past dispute decisions. First, unlike for international trade, Sapiro (2015) points out that there is no single IIA protecting foreign investors and no single multilateral institution that governs international investment policy or arbitration [57]. Consequently, governments have developed investment law on an ad hoc basis from which customary international law has emerged [57]. Additionally, a number of articles highlighted that the interpretation of relatively vague IIA obligations is left to the discretion of each arbitration panel [17, 53, 57] and panels are not obligated to base their decisions on previous dispute decisions [13, 17, 58]. This leads to different interpretations of the law and different assessment of cases involving the same facts, in turn generating competing case law which provides the basis for future tribunals to continue to reach different conclusions in almost identical cases [58, 60, 63]. For example, Johnson (2017) points out that while Australia’s win on jurisdiction made it more politically viable for other states to also introduce similar standardized tobacco packaging regulations, future tribunals are not required to follow previous decisions, and therefore tobacco companies may pursue investment arbitration for similar regulations elsewhere [69]. Various analyses also argued that uncertainty may be compounded by the lack of an appeal mechanism in investment arbitration through which parties can seek review of the interpretation of a law [16, 60, 63, 69, 72]. Further, investment arbitration proceedings have historically been criticised for a “lack of transparency and access” [17]. A number of analyses suggested that a combination of these factors make it challenging for governments to evaluate the compliance of a proposed regulation with their investment obligations, creating uncertainty that may contribute to a chilling effect on certain public health-relevant regulatory development [16, 58, 67].

#### Cost of investment arbitration

The high costs associated with defending against an investor’s claim may also make investment arbitration a powerful tool for THCCs to generate a chilling effect on domestic health regulation, particularly in the context of inequitable financial resources between corporations and states. While arbitration panels don’t have the authority to order the reversal of regulations, they can award monetary compensation to investors covering the estimated damage resulting from expropriation and loss of profits [53]. In their analysis of investor compensation in ISDS cases, Bonnitcha and Brewin (2020) point out that countries tend to face exaggerated investor claims for such damages with actual awards averaging roughly 40% of the amount originally claimed [73]. Actual awards averaged US$250 million between 2010 and 2020 and there are approximately 50 known cases of awards over USD 100 million, including eight cases where awards were more than USD 1 billion [73]. The largest individual award of compensation to date is USD 40 billion awarded in Hulley vs. Russia (the largest of several related claims arising out of the nationalization of the Yukos Oil Company, in which a total of USD 50 billion was awarded) [73]. Other studies report that even if a government is confident that they can win a dispute, experienced investment lawyers cost millions regardless of the outcome [72, 74, 75]. The literature widely reported that on average an investment arbitration case costs upwards of US$8 million for a defending state, of which legal fees account for approximately 80% [60, 63, 69, 71, 76, 77]. Jarman (2019) reported that in Phillip Morris Vs Uruguay the defendants incurred expenses of US$28.5 million however this was reimbursed when the tribunal dismissed the claims [17]. Various analyses suggest that the chilling effect on a regulation due to the cost of arbitration may occur regardless of a government’s perception of their ability to successfully defend proposed legislation [13, 52, 55, 63, 69, 78].

Only anecdotal evidence was identified supporting the theory that high cost of investment arbitration can contribute to regulatory chill. Three included articles reported that Uruguay’s government acknowledged it was only able to defend itself against Phillip Morris International’s challenge after receiving support from Bloomberg Philanthropies to finance their legal team [13, 61, 79]. In 2002, Indonesia granted exemptions from an open-pit mining ban in protected forest areas to a list of 13 companies after they threatened to initiate arbitration against the state [16, 67]. Shekhar (2016) reported that the Indonesian government claimed they did not have the finances to pay the compensation to investors [67].

#### Biased arbitration panels

Uncertainty in predicting the outcome of investor-state disputes may also be exacerbated by both explicit and implicit bias (real or perceived) in arbitral rulings [80]. Arbitration panels are composed of three arbitrators, each party appoints one and the third is jointly appointed. Each arbitrator is compensated by parties to the dispute on a case-by-case basis without secure tenure. A number of articles/reports included in this review identified concerns over potential arbitrator conflict of interest, the independence of arbitrators and explicit bias created by the system [57, 58, 65, 81]. Hawkins and Holden (2016) argue that if an arbitrator interprets provisions in such a manner as to favour investors, they may promote future use of the ISDS mechanism by foreign investors [12] and others suggest investors may be more likely to appoint that arbitrator in future arbitration [16, 63]. Conversely, if an arbitrator develops a reputation for ruling in favour of states, they may be more likely to be repeatedly appointed by defending states. Additionally, Langford and colleagues (2017) point out that arbitral appointment is heavily influenced by legal counsel which creates potential for special favours [82].

A number of analyses suggest implicit bias in the arbitration system may arise since lawyers are free to rotate between roles as legal counsel and arbitrators in different cases [57, 63, 72]. This so-called ‘double hatting’ has been empirically confirmed to have persisted over time amongst a very small elite group of actors [82]. Double-hatting was mentioned as problematic in two articles since when making a decision as arbitrator in one dispute, it may be difficult not to be influenced (consciously or subconsciously) by the arguments made as counsel in another dispute with similar legal issues [72, 82]. Further, a number of analyses point out that arbitrators are experienced international trade and investment lawyers, nearly all from HICs and typically have no expertise in public policy which may contribute to an implicit bias towards foreign investors [16, 55, 63]. A number of arbitrators have also served as members of boards of multinational corporations creating significant conflict of interest issues [60].

We identified mixed empirical evidence for bias in arbitral rulings. One 2016 analysis of 197 ISDS cases involving LMIC defendants from the UNCTAD database before September 2016 found that arbitral panels overall were not more likely to rule in favour of the claimant with a ratio of investor wins over state wins of 0.89 [83]. The study did find evidence that panels identified a priori as biased towards investors were more likely to rule against LMIC respondents but the same biased rulings were not found in disputes involving HICs [83]. Another empirical legal analysis of trends in legal interpretation in ISDS cases conducted in 2012 found tentative evidence that arbitrators favour investor claimants over respondent states [84]. Regardless of whether this bias is real or simply perceived, it may nonetheless create uncertainty for governments in determining the likely outcome of a potential dispute and may therefore contribute to a regulatory chilling effect.

Notably, the high revenue gained through investment arbitration perversely incentivises law firms to promote the use of investment arbitration and the rise in investment arbitration annually since the 1990s has, in part, been attributed to such promotion [85]. Arbitrators have also actively promoted the importance of the ISDS mechanism to promote foreign investment and has lobbied against reform [60, 86].

### Evidence of regulatory chill

#### Response chill

Response chill refers to a chilling effect on a specific proposed or adopted regulatory measure after a government becomes aware of the risk of investment arbitration [12–14]. This can result from the actual initiation of a dispute, threat of an impending dispute, or perceived threat based on other states’ experience in relation to similar legislation [71]. Evidence for public health regulation response chill was primarily identified in individual case-studies. Curran and Eckhardt (2017) reported that in the 1990s, the tobacco industry used NAFTA’s Investment Chapter 11 to argue that Canada’s proposed tobacco plain packaging regulation equated to illegal expropriation of its trademark requiring hundreds of millions of dollars in compensation [87]. Threats of investment arbitration heavily impacted the Canadian parliament’s decision to abandon the proposal [87]. Sud and colleagues (2015) reported that in the early 2000s a ban on misleading cigarette labelling terms (e.g. ‘light’ and ‘mild’) was also delayed in Canada after Phillip Morris again argued the ban violated NAFTA [88].

Two sources reported that in 2014, threats of investment arbitration by the tobacco industry over New Zealand’s proposed plain packaging bill was a key reason adoption of the bill was delayed [89, 90]. BAT New Zealand, for example, claimed the bill was in violation of New Zealand’s IIA obligations and would entitle the company to "an arbitral award requiring New Zealand to repeal the legislation and/or pay substantial sums in compensation" [90, 91]. It is also relatively widely claimed in the literature that New Zealand and Thailand also delayed progress on standardized packaging until the decision was known in the investor-state case against Australia [18, 53, 67, 78, 89]. Gruszczynski (2014) identified this case as one of various factors that delayed the EU’s draft Tobacco Products Directive and the UK’s 2014 decision not to proceed with plain packaging [53]. Tam and van Walbeek (2014) reported that, in 2012, BAT attempted to use the Australian legal cases to intimidate Namibian officials into not proceeding with their proposed Tobacco Products Control Act [92].

It is important to note that Australia pursued tobacco standardized packaging legislation despite industry arguing, among other things, that the measure would violate their intellectual property rights, expropriate their investment and not accord them fair and equitable treatment under the Australia-Hong Kong Bilateral Investment Treaty [93]. This indicates that the threat of an investor-state dispute is just one of multiple interacting factors influencing policy decisions.

#### Precedential chill

The potential for state actors to change a regulation in response to a settled or resolved investor-state dispute due to concern of future arbitrations based on the same (or similar) regulation, has been defined as precedential chill [67, 71]. In this case a state will roll-back progressive public health policy after they or another country loses/settles an investor-state dispute [71]. One example that may to some extent reflect this form of regulatory chill is the 1997 ISDS case of US Ethyl Corporation vs Canada as reported by Tietje (2014). Ethyl Corporation, a US company importing and distributing the gasoline additive MMT in Canada, brought an investment claim against the Canadian government under NAFTA for banning MMT imports and inter-provincial trade [71]. After a number of Canadian provinces successfully challenged the legitimacy of the MMT ban in domestic courts, the Canadian government decided to settle the ongoing NAFTA investment dispute by agreeing to pay Ethyl CAD$20 million and repealed the MMT ban [71]. While some consider the loss in domestic courts was the primary driver of the Canadian government’s decision to roll-back the ban, others argue investment dispute-related concerns may have also played a role [71]. In 2011, Lone Pine Resources initiated a dispute against Quebec’s revocation of its right to mine for oil and gas under the St. Lawrence River without compensation after a moratorium on hydraulic fracking of shale gas was passed in 2011 due to public health and environmental concerns [71]. While the outcome of the case is still pending, Tietje points out that some experts are concerned about it generating regulatory chill [71], “for example, the director of the Sierra Club’s trade program claimed in 2013 that ‘If a government is not even allowed to take a time out to study the impact without having to compensate a corporation, it puts a tremendous chill on a governments’ ability to regulate in the public interest.” [71, 94].

#### Anticipatory chill

Some researchers have raised the concern of anticipatory chill- that policy-makers may take into account potential disputes with foreign investors during the policy development process, hampering regulatory progress across a range of public health policy areas [67, 71, 95]. Côté’s 2014 study assessed 50 in-depth interviews complemented by an electronic survey of 114 of Canadian health and safety and environmental regulators concluded there was low level awareness among policymakers of the potential threat of investor-state challenges to regulations [76]. The study also found that policymakers rarely considered Canada’s trade and investment obligations when developing regulations, but when they did, WTO obligations were of primary concern [76]. Additionally, the study included in-depth interviews with tobacco control regulators from 11 countries complemented by 28 electronic surveys completed by tobacco control regulators in 28 different countries [76]. The findings here reflected those found amongst Canadian regulators.

Somewhat in contrast, Van Harten and Scott’s 2016 Canadian case study including 52 key informant interviews identified that the Ethyl Corp case described above “drew much more attention to ISDS” and after which there was widespread reluctance to develop policy since it might also trigger litigation under NAFTA [96]. The study found that various “government ministries have changed their decision-making to account for trade concerns including ISDS” [96]. This included a standardized regulatory impact assessment process for evaluating policy and regulatory proposals for trade and ISDS risk and generally significantly greater involvement of government lawyers to vet proposals for compliance with trade and investment rules [96].

Other analyses also reported that internal vetting processes for compliance with international trade and investment obligations had been institutionalized in multiple other countries. For example Peru, Guatemala, Panama and the Dominican Republic have adopted formal vetting processes for any new regulation to consider its trade and investment implications including a ‘dispute prevention’ mechanism [13]. New Zealand and Australia have adopted regulatory management regimes that incorporate risk assessment processes assessing policy consistency with international trade and investment obligations [90]. The Regulatory Coherence chapter in the CPTPP encourages parties to establish regulatory impact assessments to ensure regulations are necessary, not unacceptably costly, and evidence-based; a national body for ensuring inter-departmental consultation and coordination; and establishes a Committee on Regulatory Coherence comprising of government officials to promote regulatory coherence between CPTPP parties [97]. The institutionalization of such mechanisms has the potential to shift health policy decision-making power from departments of health to departments of trade or state legal actors from an early stage of policy development.

### Contextual factors

Contextual factors were not a primary unit of analysis in any of the literature included in this review. However, we identified a number of contextual factors, primarily within case study analyses, that may mediate the ability of THCCs to promote investment-related policy non-decisions or ISDS-related regulatory chill. In this section we tentatively outline these factors and present a synthesis of the supporting evidence. It was not possible to assess the potency of each factor and generally a combination of factors are likely to be influencing any single policy/regulatory decision.

#### Domestic economic conditions

Poorer economic conditions and limited domestic finance were of the more commonly mentioned factors in the literature that may increase the likelihood of regulatory chill. Behn and colleagues (2018) argue that as ‘rule takers’ in trade and investment negotiations (due to their relatively weaker economies), LMICs may be committed to a more diverse and inconsistent set of investment rules compared to HICs that tend to be ‘rule makers’ and therefore able to ensure their IIAs are relatively uniform in content [80]. This combined with Bernasconi-Osterwalder and colleagues (2012) point that with limited financial and technical resources to ensure their regulatory regimes comply with strict and demanding investment protection standards set out in IIAs, LMICs may be particularly aware of their vulnerability to legal threats from investors in relation to regulatory development [72] and therefore reticent to implement regulatory change even before a threat of investment arbitration is made [72].

A number of analyses raised points that tended to support the theory that with limited government budgets, LMICs may also be likely to perceive the potential costs associated with an investor-state dispute as unacceptably high. Investor-state disputes typically cost more than US$1 million annually which would exceed the budget for tobacco control in many LMICs [61]. In a ruling against the Czech Republic, an arbitration panel ordered compensation equivalent to the country’s annual health budget [12, 58] and in a case against Ecuador investors claimed the equivalent of 7.5% of annual government expenditure and was eventually awarded 1.92% of it, greater than government’s annual expenditure on health [72]. Legal expenses can affect the cost-benefit analysis of a health measure by increasing the initial costs [49]. Considering these high costs, governments, and particularly LMICs, may be more likely to take a risk averse approach and refrain from regulating [49, 79, 95].

Level of dependence on foreign investment was also a contextual factor identified across several analyses. Two analyses argued that LMICs with major concerns over unemployment, public and private debt and the need for economic growth may be more dependent on foreign investment and therefore may be particularly vulnerable to investment-related policy non-decisions [9, 81]. Others made related arguments that some countries may be particularly concerned that NCD prevention regulations will affect investors’ profits and/or trigger an investor-state dispute resulting in the government being perceived as ‘anti-investor’, potentially leading to investor flight or detracting future foreign investment [16, 89]. One identified example that may support this theory is that in 2002, in the context of a relatively weak economy and reliance on the extractive industry, Indonesia backed down on a proposed ban of open-pit mining after mining companies threatened investment arbitration [16, 67]. Brown (2013) also argues that reliance on foreign aid may make some LMICs more vulnerable to investment arbitration-induced regulatory chill since they may not wish to negatively affect their relationship with an investor’s home state which may be an important source of financial aid [16].

#### Domestic technical resources

Without internal legal expertise in international investment law or the finances to hire expensive international lawyers, regulators, again particularly in LMICs, may find it difficult to reasonably evaluate their compliance with their international investment obligations. This may result in THCC threats being perceived as more credible than they actually are. As such, legal capacity constraints may reduce the political will required for implementation of a risk commodity regulation [49]. Further, understanding their defense would potentially be sub-optimal, may increase the likelihood that LMICs decides to refrain from regulating. While we found no specific evidence to support this theory, two recent studies of investor-state dispute decisions found tentative empirical evidence that developing countries are much more likely to lose a dispute than developed countries [80, 84]. At the same time, a THCC may presume they would have an advantage in litigation due to their superior legal support and may therefore be more likely to bring weak claims against LMICs [80].

#### Political conditions

Two articles mentioned the potential for political orientation to affect the likelihood of regulatory chill [23, 81]. Crosbie and Thomson (2019) provide the example of standardized tobacco packaging where they observe the “case of New Zealand was similar to the UK where the centre-right party leadership was cautious, and delayed enacting standardized packaging [once aware of the ISDS case against Australia]. In contrast, the centre-left and left leadership in Australia and Uruguay respectively, was bold in rejecting the industry legal threats from the outset” [98]. Notably, some Australian right-wing parties opposed to standardized packaging used the legal risk of introducing such legislation as a reason not to regulate [89].

Tietje (2014) argues that policy central to a government’s mandate seems to be more likely to withstand legal threats from industry [71]. Taken together, a number of analyses provided examples supporting this- while the New Zealand government’s position on standardized packaging had been ambivalent before Phillip Morris initiated a dispute with Australia [90], tobacco control was a key part of the government of Uruguay’s policy plan [21] and proved critical to withstanding industry legal threats and eventual investment arbitration [99]. Similarly, in Australia there was strong bipartisan support for tobacco control [89]. In addition, policy champions/political leadership is crucial, as was also seen in the Australia standardized packaging case [89].

Precedents set by other countries and reputational concerns appear to also provide a counter force to fear of investment litigation. For example, after more than a 6 year delay due to concerns over investment arbitration [89], New Zealand’s government brought plain packaging into force in 2018, at least in part to avoid reputational damage in light of Australia’s progressive approach to tobacco control [90].

#### Previous exposure to investment arbitration

High levels of awareness or previous experience with investor-state disputes may also make a government especially reluctant to engage in a dispute [95]. However, the very limited evidence relevant to this theory is mixed. Van Harten and Scott’s 2016 Canadian case study identified that concerns of investment litigation over new regulations was more pronounced after a ministry had been involved in a NAFTA dispute [96]. Weiss (2013) described a contrasting outcome relating to legislative measures introduced in Slovakia that partly reversed the previous privatization of the Slovak health insurance market. After initiating an investor-state dispute in 2008 over these measures, Achmea B. V, an investor in the private health insurance sector, was awarded EUR22 million in compensation by an arbitration panel in 2012 [60]. In 2009 the government faced a similar investor-state dispute initiated by the investor HICEE B. V [60]. These cases however did not appear to prevent the Slovak government from progressing on legislation to establish a public health insurance scheme. Further, the government did not abandon this proposal when faced with yet another investor-state dispute initiated by Achmea in 2013 claiming expropriation of its stake in a Slovak health insurance company. This is evidenced by the state’s decision to proceed to investment arbitration, which they eventually won in 2014 [100]. Notably, the Slovak Republic repeatedly challenged the 2012 arbitral award to Achmea, first losing in the German courts in 2016, but ultimately prevailing in the European Court of Justice which set aside the award in 2018 [101]. This relatively unprecedented legal ruling may have since increased Slovakia’s (and potentially other countries’) confidence to pursue similar public health legislation despite ISDS threats/proceedings.

#### Industry-related factors

Some evidence synthesized across different analyses indicates that industry legitimacy may play a role in determining the likelihood of regulatory chill. During the 1990s when the tobacco industry still maintained a degree of legitimacy and political support, its threats of an investor-state dispute against Australia and Canada over proposed standardized tobacco packaging were highly effective and contributed to the regulations being abandoned [15, 102]. Twenty years later, when the tobacco industry had lost political capital and been denormalized, similar threats and pursuit of investment arbitration against Australia over similar legislation, did not produce the same direct effect. Johnson (2017) remarks that currently the UPF and alcohol industries are perceived by governments as important stakeholders in growing national economies and to address NCDs, and their products are perceived by the public as not necessarily harmful to health if consumed in moderation [69]. While it remains to be seen, investment arbitration may therefore be a powerful instrument available to these industries to prevent or stall food and alcohol regulatory development. That said, high levels of industry legitimacy may mean these industries do not need to resort to legal threats since they have multiple other effective tools at their disposal to influence the regulatory environment.

Thow and colleagues (2015) point out that the real or perceived economic contribution of an investor may determine their influence in policy decisions [81] and, we suggest, possibly also their ability to elicit regulatory chill using the ISDS mechanism. Specific evidence to support this theory was, however not identified.

#### Risk commodity-related factors

Regulation of alcohol and unhealthy food (to reduce consumption) is not supported by an international treaty and there is relatively limited availability and acceptability of scientific evidence for policies to reduce alcohol abuse or unhealthy diets [69, 103]. There is also a far broader range of alcohol and unhealthy food products with differing compositions, and low consumption of these products does not necessarily have demonstrable harmful health impacts [69]. For these reasons, it may be relatively more difficult for host states to prove that food or alcohol regulatory measures are proportionate and contribute to legitimate public health objectives [69]. We suggest these factors may also make governments more susceptible to ISDS-related policy chill, although again we identified no evidence to support this theory.

### Recommendations

Given the described drivers of international investment-related policy non-decisions including regulatory chill, and with consideration of modifiable contextual factors, various potential recommendations were also identified through analysis and synthesis of existing literature.

#### Addressing the political and economic drivers of investment-related policy non-decisions

Upstream strategies that prevent or at least limit ‘unhealthy’ investment may be one option for curtailing THCC’s influence in NCD policy processes. These include adopting health-orientated conditionalities on FDI by THCCs for example on fiscal issues, marketing, product composition and labelling [104]. Other upstream options include compulsory health risk assessments for evaluating proposed incentives for FDI in relevant sectors [4]. However, unless efforts do not also focus on shifting the dominant belief amongst powerful economic actors that employment and economic growth achieved largely via market strategies will address NCDs, it is unlikely governments will be willing to consider restricting FDI on health grounds.

International health instruments may be used to shift towards ‘healthier’ investment policy norms. These could include ensuring THCCs are on a list of sectors excluded from further investment liberalisation and encouraging countries, in a non-discriminatory manner, not to promote or allow any further investment from THCCs unless certain health conditionalities are met [38]. International health instruments may also be used to promote the protection of health regulatory space in IIAs [17]. For example, guidelines on controlling tobacco investments (both foreign and domestic) could be incorporated within the FCTC [38].

Limiting investing THCCs’ ability to leverage their economic contribution to influence domestic health policy will also require transparent and enforceable rules governing interactions between THCCs and governments [105]. Article 5.3 of the FCTC, for example, legally obligates parties to adopt measures that protect "their public health policies related to tobacco control from commercial and other vested interests of the tobacco industry" [106].

#### Reforming international investment protection rules and procedures

Various strategies for reforming international investment rules that enable THCCs already invested in a country to use the ISDS mechanism for generating regulatory chill have also been proposed [49]. The first option is to simply exclude the ISDS mechanism from IIAs. A number of countries have already taken assertive action on this. For example, a number of South American countries, South Africa, and Indonesia having either refused to permit the inclusion of ISDS in new TIAs or cancelled/let lapse existing TIAs containing ISDS [107]; Brazil has concluded a number of Co-operation and Facilitation Investment Agreements which exclude ISDS altogether [108]; and 28 EU states have agreed to terminate ISDS arrangements between themselves, committed to exclude ISDS from any of its current negotiations, proposed replacing ISDS with an Investment Court System modelled after the WTO dispute resolution system with appointed permanent judges and an appellate mechanism [109].

Including general health exceptions in future IIAs and investment chapters in trade agreements is one of many ‘softer’ options. However, it may do little to reduce litigation bought by corporations since it still requires states to provide an affirmative defense [61] and some legal analysts argue that protection of investor rights usually trumps provisions protecting health policy space [66]. For example, although NAFTA’s Investment Chapter contained provisions affirming the right of governments to protect public health, the measures needed to be "otherwise consistent with this Chapter", essentially rendering the public health protection article redundant [66]. Similarly worded exceptions are included in the investment chapters of the recently signed agreement between Canada and the EU (CETA) [17]. The CPTPP (and previously drafted TPPA) include in Annex 9.B that "non-discriminatory regulations … designed for legitimate public welfare objectives", including health and the environment, "do not constitute indirect expropriation, except in rare circumstances" [64]. This seems to protect regulatory space against disputes based on indirect expropriation [75]. However, as others have pointed out, without defining ‘rare circumstances’ or what constitutes a ‘legitimate public welfare objective’, interpretation remains somewhat open [52, 61, 75, 86] and public health measures including tobacco, alcohol and food regulations could still be considered as compensable indirect expropriation [52].

Complete carve outs (or exclusions) for specific areas of investment from IIA obligations is another rules-based option [110]. A specific carve-out of tobacco control measure is included in the CPTPP text [64], although there are concerns this may undermine the protection of other health areas under general health exceptions and an overall regulatory carve-out or strengthening of the general exception may be a better approach [111]. For example the Peru-Australia Free Trade Agreement clarifies that ‘no claim may be brought under this Section [ISDS] in relation to a measure that is designed and implemented to protect or promote public health’ [112, 113]. While this may be possible for tobacco, for both political and issue complexity reasons, it may not be possible to do the same for alcohol and UPFs [4, 111].

Clarifying the meaning of key terms/obligations to limit the use of ISDS by investors and interpretation by arbitral panels is also needed. For example one strategy to clarify foreign investor’s ‘legitimate expectations’ could be to develop a national policy for products harmful to health clarifying that foreign investors cannot reasonably expect the host country not to progressively advance public health measures in these areas [4]. In the CPTPP’s Investment Chapter the Fair and Equitable Treatment Provision clarifies that an investor’s expectation’ by itself is insufficient grounds on which to sue for loss or damages [64]. The CPTPP’s (and previous draft TPPA’s) Investment Chapter also attempts to clarify the meaning and restrict the application of indirect expropriation. Annex 9-B refers to indirect expropriation as "an action or series of actions by a Party [that] has an effect equivalent to direct expropriation without formal transfer of title or outright seizure" [64, 86]. A footnote to Annex 9B attempts to clarify that "[f] or greater certainty, whether an investor’s investment-backed expectations are reasonable depends, to the extent relevant, on factors such as whether the government provided the investor with binding written assurances and the nature and extent of governmental regulation or the potential for government regulation in the relevant sector" [64, 86].

Procedural improvements to the ISDS mechanism should also be sought to reduce the exposure of governments to ISDS and the uncertainty they face in relation to potential investment arbitration. These could include requiring investors to first exhaust domestic courts before proceeding to an international tribunal; giving the states involved in a dispute the right to issue binding interpretations of ISDS provisions; making it easier to dismiss frivolous claims; increasing openness and transparency of proceedings; asserting the right for a state to deny an investor protection under an IIA if they fail to comply with their obligations, which should include the human right to health; allowing states to file a counterclaim in response to a primary investor dispute filed by an investor for any violations of their obligations; and prohibiting arbitrators from working as lawyers on investment disputes [57, 114]. As proposed by the EU, establishing a permanent international investment court system with tenured judges and an appeal process could promote the development of a "more coherent body of jurisprudence on substantive and procedural international investment law" and eliminate the potential bias of ad-hoc arbitration panels [57]. Adopting a ‘loser pays’ principle could also help prevent regulatory chill directly associated with the high cost of investment arbitration.

#### Post-treaty adoption strategies to reduce the risk of regulatory chill

Where policymakers lack understanding of their country’s investment protection obligations, threat of an investment dispute, or desire to avoid such a threat, may generate regulatory chill, even when a health regulation is in fact IIA compliant. Downstream, post-treaty adoption strategies to reduce the risk of regulatory chill include, therefore, close collaboration between and capacity-building within health, trade, and legal departments to ensure policymakers can recognise spurious ISDS threats and are confident in pursuing health regulations that are already IIA compliant. Given the complexity of many countries’ IIA obligations, ongoing specialist legal advice is also essential to maintain government confidence after threats of an investor-state dispute. In Australia’s standardized packaging case, legal scholars provided sound legal advice to the government emphasizing their right to adopt this regulation [89]. External technical and financial support will be important for many LMICs facing investment arbitration as was observed when Uruguay’s government made the decision to defend its tobacco health warning labels regulation in an investor-state dispute only after receiving external support [99].

Additionally, developing capacity within health departments to design regulations that effectively address public health objectives but are also IIA-compliant, reduces the risk of a successful ISDS challenge. Again, this requires close collaboration between health, trade and legal departments both before and throughout the policy development process. Collaboration of this nature was observed in Canada and Brazil when developing their tobacco additives bans and in Australia during the development of their standardized packaging regulation to ensure compliance with their trade and investment obligations and to pre-empt industry opposition [89, 102, 103]. However, this level of cooperation between trade and health actors was only considered possible when export interests were not an issue [103]. Further, it is important to assert that health regulations should be designed primarily to protect and promote health with minimizing the risk of an investment (or trade) challenge a secondary concern. We acknowledge however, that close collaboration between health and trade departments may have the potential to contribute to an anticipatory chilling effect on health policy where policy effectiveness is diluted to ensure IIA compliance.

International health instruments, particularly legally binding agreements, may give governments the confidence to pursue public health regulations despite threats of investment arbitration. The FCTC again played a clear role in prompting Australia to adopt standardized packaging legislation and gave the government confidence it could withstand an investor-state dispute initiated by Phillip Morris [89]. Further, some legal experts argue a consistent and recurring use of the FCTC by trade and investment dispute tribunals has resulted in the normative integration of the FCTC into the investor-state dispute process [115], which may also contribute to governments’ future confidence in adopting FCTC regulations.

Mobilization of transnational public health advocacy networks that include a wide range of actors, has been critical in promoting policy action despite the risk of legal action [23, 66, 81, 89, 99, 102]. During Australia’s ISDS case over its standardized tobacco packaging legislation, a tobacco control advocacy network extending well beyond the health sector that had built close trusting relationships over the past decade with government officials, policymakers and the media which gave them access to and influence within decision-making spaces at that crucial time [99]. In Van Harten and Scott’s 2016 Canadian case study, a number of stakeholders also identified public health and environmental group support as important for preventing regulatory chill in response to a threatened investor claim under NAFTA over a proposed ban on cosmetic use of pesticides [96]. Similarly in the Pac Rim mining companies case against El Salvador, local community groups organized and pressured the government not to approve the mine for the protection of the local communities’ health and the environment [16], this may have contributed to the government’s decision to proceed to arbitration which was ultimately decided in their favour.

Strategies used by advocacy networks to reduce regulatory chill also include influencing issue interpretation through dissemination of strategic framing. Australia’s tobacco control advocates consistently framed standardized packaging as beneficial both economically and for public health, highlighted the unique harms of tobacco and the child protection imperative [89]. They also emphasized Australia’s international legal commitment to the FCTC and exposed the patterns of manipulative industry legal attacks [89]. Importantly, they avoided engaging in debates about claimed investment treaty breaches and advised politicians to do the same. Together these efforts were credited by health policymakers as important contributors to avoiding regulatory chill [89].

## Discussion

This review found that THCCs may be incentivised to threaten or pursue an investor-state dispute since the costs may be outweighed by the benefits (even of simply delaying regulatory adoption) and since this effect tends to ripple out across jurisdictions. Drivers of regulatory chill may include ambiguity in treaty terms, inconsistency in arbitral rulings, potential arbitrator bias and the high cost of arbitration. While THCCs have recently received unfavourable outcomes in investor-state disputes relating to tobacco control regulation, evidence indicates ISDS can make innovation costly for governments and delay policy adoption both within the country directly involved but also in other jurisdictions. Additionally, governments are taking pre-emptive action for example by adopting standard assessments of public health regulatory proposals for trade and investment dispute risk. Various economic, political and industry-related factors likely interact to increase (or decrease) the ultimate risk of regulatory chill.

While regulatory chill-related analysis is an emerging area of research, comparatively very limited empirical research primarily analysing the broader political and economic dimensions of international investment-related NCD policy non-decisions was identified. However, there was some case study evidence indicating that THCCs do take advantage of governments’ prioritization of foreign investment over NCD prevention objectives to influence the NCD prevention regulatory environment.

Over the longer term, promoting the adoption of many of the recommendations outlined in this review requires that public health policymakers vastly increase their capacity to actively engage with investment policy development and agreement negotiations. While scholars have advocated for departmental capacity-building through technical training on the linkages between trade and health, and coordination between trade and health departments [99, 102, 116–118], evidence presented in this review indicates such processes may be equally important to promote health objectives in investment policymaking.

Driving policy change will also require health advocates adopt discursive strategies that promote a shift in the way FDI, THCCs and NCDs are perceived, particularly by dominant economic actors and the public. Shaping perceptions of industry legitimacy through actor and issue framing is one such strategy. Tobacco control advocates have illustrated that industry delegitimization (e.g. by exposing unhealthy and nefarious industry practices, emphasizing the public health, social and economic burden of health harmful products, and de-normalizing THCCs) can help shift perceptions and ultimately policymaking norms towards prioritizing health over foreign investment and excluding investors from health policy decision-making spaces [17, 89]. This includes by enshrining these norms within both international health and investment agreements. Notably though, in a number of tobacco-producing countries, the economic imperative remains dominant and industry influence substantial. Further, due to issue complexity, shifting perceptions and norms relating to UPFs and alcohol regulation will be an even greater challenge. Norm-shifting may also increase the likelihood that a government will withstand threats of ISDS from THCCs [89] and investment arbitration panels may be influenced by such norms in their rulings. For example, in the Uruguay case the panel commented that several modern IIAs explain nondiscriminatory regulations with ‘legitimate public welfare objectives’ like public health, do not constitute indirect expropriation [119].

## Limitations

This review has a number of important limitations. Restricting the review to sources published after 2008 and our limited capacity to undertake multiple secondary iterative literature searches in keeping with the realist approach, may have resulted in relevant explanatory mechanisms and data that supported or challenged them, not being captured by this review. Also, identification and development of explanatory mechanisms may have been limited due to the very few studies identified on investment and health policy that explicitly engaged with political economy theory.

## Conclusions

This review finds some evidence of the real potential risk of NCD prevention regulatory chill and tentatively suggests the contexts in which it may be more likely to occur. However, international investment-related NCD prevention policy non-decisions driven by broader political and economic factors may well be more widespread and restrictive of NCD prevention regulatory environments. As such, there is a clear need for research that explores the more political and economic dimensions of how international investment liberalization may shape actor interests and priorities in ways that affect NCD policy. This will require empirical studies that engage with theories and approaches from international relations and political science, including political economy and power analyses.

The findings of this review indicate the need for a broader research agenda on the implications of foreign investment on NCD policy and objectives. Such an agenda should include regulatory chill-specific questions already posed by others [23, 120] such as how does the perceived risk of an investor-state dispute or direct challenge by foreign investors affect health policymaker’s decisions?; but also questions that investigate the broader political and economic drivers of investment-related NCD policy non-decisions, for example, what are the barriers to greater coherence between investment and NCD policy and objectives?

## Supplementary Information

**Additional file 1.**

**Additional file 2.**

**Additional file 3.**

## Data Availability

Not applicable.
