# Supplementary material for: International investment liberalization, transnational corporations and NCD prevention policy non-decisions: a realist review on the political economy of tobacco, alcohol and ultra-processed food
Source: Global Health. 2021 Nov 24;17:134. doi: 10.1186/s12992-021-00784-3 (PMC8611909; doi:10.1186/s12992-021-00784-3)
Supplement: Supplementary file 1 — Additional file 1. [file 12992_2021_784_MOESM1_ESM.docx]

**Supplementary Text I:** **Search concepts and terms**

| **Concept** | **Terms** |
| --- | --- |
| 1: Trade | "international trade" OR "trade agreement*" OR "trade rule*" OR "trade law*" OR "trade polic*" OR "trade and investment polic*" OR "foreign direct investment" OR "foreign investment" OR "international investment" OR "investment treat*" OR "investor state dispute settlement*" OR "technical barriers to trade" OR "trans-pacific partnership agreement" OR "regional comprehensive economic agreement" OR "transatlantic trade and investment partnership" OR WTO OR "World trade organi#ation" |
| 2. Policy/regulatory chill | "Policy chill*" OR "policy freeze" OR "regulatory chill*" OR "regulatory freeze" OR "chilling effect" OR "non-decision making" OR "policy space" OR "regulatory space" OR "regulatory constrain*" OR "policy constrain*" OR "regulatory delay" OR "policy delay" OR "regulatory revers*" OR "policy revers*" |
| 3: Nutrition security | nutrition OR "food polic*" OR "food regulation*" OR "food and beverage regulation*" OR "food labelling" OR "breast milk substitute*" OR "infant formula*" OR "food industry" |
| 4: Tobacco control | "tobacco control" OR "tobacco polic*" OR "tobacco regulation*" OR "smoke-free polic*" OR "smoking prevention" OR "tobacco industry" |
| 5: Alcohol regulation | "Alcohol polic*" OR "alcohol regulation*" OR "alcohol labelling" OR "alcohol industry" |
| 6: Food, tobacco and alcohol trans-national corporations | "food industry" OR "tobacco industry" OR "alcohol industry" OR corporat* |
| 7. Policy process | Policy ADJ1 (formulation OR making OR process OR development) OR governance |
| 8. Trade-sensitive health-relevant policy areas | "health polic*" OR "tobacco control polic*" OR "tobacco regulation*" OR "alcohol polic*" OR "alcohol regulation*" "food polic*" OR "food regulation*" OR "nutrition polic*" |
| Search strategies:  1: Title OR Abstract ((1 OR 2) AND (3 OR 4 OR 5))  2: Title OR Abstract (6 AND 7 AND 8) |  |
