# Supplementary material for: International investment liberalization, transnational corporations and NCD prevention policy non-decisions: a realist review on the political economy of tobacco, alcohol and ultra-processed food
Source: Global Health. 2021 Nov 24;17:134. doi: 10.1186/s12992-021-00784-3 (PMC8611909; doi:10.1186/s12992-021-00784-3)
Supplement: Supplementary file 2 — Additional file 2. [file 12992_2021_784_MOESM2_ESM.docx]

**Supplementary Text II: Screening Tool**

| **Author, title of publication (year):** | **Type of study/source** | **Method stated** | **Data source stated (or referenced)** | **Empirical research** | **Policy area** | **Inclusion/Exclusion criteria: Relevant** | **Inclusion/Exclusion criteria: Reliable** | **Decision to include/exclude?** | **Reason for decision** | **Full text accessible** |
| --- | --- | --- | --- | --- | --- | --- | --- | --- | --- | --- |
|  |  |  |  |  |  |  |  |  |  |  |
|  |  |  |  |  |  |  |  |  |  |  |
|  |  |  |  |  |  |  |  |  |  |  |
