# Supplementary material for: International investment liberalization, transnational corporations and NCD prevention policy non-decisions: a realist review on the political economy of tobacco, alcohol and ultra-processed food
Source: Global Health. 2021 Nov 24;17:134. doi: 10.1186/s12992-021-00784-3 (PMC8611909; doi:10.1186/s12992-021-00784-3)
Supplement: Supplementary file 3 — Additional file 3. [file 12992_2021_784_MOESM3_ESM.docx]

**Supplementary Text III: International investment agreement terms and obligations relevant to regulatory chill**

While a number of IIA terms and obligations are important, we focus here on those that have featured most prominently in analyses of IIAs for their potential to restrict health harmful product regulatory space; or used by investors as the grounds on which to pursue an investor-state dispute.

**Definition of an investment**

The majority of IIAs define investment broadly, including ‘every asset that an investor owns or controls, directly or indirectly, that has the characteristics of an investment, including such characteristics as the commitment of capital or other resources, the expectation of gain or profit, or the assumption of risk’ (1, 2). This definition is generally followed by forms the investment could take an usually explicitly include intellectual property rights and other ‘tangible or intangible, movable or immovable property, and related property rights, such as leases, mortgages, liens, and pledges’ (1).

**Prohibition of expropriation**

While direct expropriation is relatively clearly defined as the direct taking by the state of an investor’s property (3), in recent decades indirect expropriation has been one of the most commonly cited grounds for initiation of an investor-state dispute by investors and has been vaguely defined in IIA texts resulting in various different interpretations of it by arbitration panels (4). Indirect expropriation is broadly considered to be regulatory action taken by a government not reasonably expected by an investor that affects the value of a company’s investment and their future profits, even if that was not the intent (5). For example, in its case against Australia’s plain packaging legislation, PMI claimed indirect expropriation- arguing that the legislation significantly reduced the value of its trademark (6) and their expectation that they could reliably use their trademark was reasonable given existing national and international trademark protections (6). Challenges may arise for governments in predicting the outcome of such a claim since tribunals have considered indirect expropriation via three different approaches: the ‘sole effects doctrine’ considers only the extent of a measure’s impact on an investment, with no consideration to the purpose of a public policy measure; the proportionality approach balances the public benefits of the measure with the impact on an investor; and the third approach carves-out certain legitimate measures of significant public value that cannot be considered expropriation regardless of their impact on an investment (7). A second element of indirect expropriation considers the extent of impact on an investment with some tribunals ordering compensation for a significant loss while others have required near total destruction of the investment value before awarding compensation (8, 9). These different approaches have been used in different arbitral awards, making it difficult for governments to predict the decision in a future potential case.

**Fair and equitable treatment**

Fair and equitable treatment (FET) is another vague concept not specifically defined in any investment treaty (4, 7, 9) yet has also been one of the most common principles on which investors have initiated an investor-state dispute and on which arbitrators have ruled (10, 11). FET has previously been interpreted by arbitration panels as entitling foreign investors to a ‘stable and predictable regulatory environment’ that protects their ‘legitimate expectations’ of profit (6, 8). In ruling on the extent of protection that should be granted, panels consider the contribution of the investment to the national economy, if the regulation is arbitrary, and the policy environment at the time the investment was made, but decisions on the scope of protection have varied significantly (7, 8, 12). For example in the Tecmed vs Mexico case the tribunal interpreted ‘legitimate expectations’ very broadly, ruling that to avoid violating their FET obligation a host state must act in such a manner as to ‘not affect the basic expectations that were taken into account by the foreign investor to make the investment;’ and that is consistent, ‘free from ambiguity and totally transparent,’ such that the investor can be aware of all the relevant rules and regulations and their respective purpose and aims before deciding to invest (7). Other tribunals have has a much narrower interpretation of FET, requiring only that states act in a manner that is not ‘egregious and shocking’ and one tribunal added that investors’ expectations ‘must be reasonable and legitimate in light of the circumstances prevailing in the host country’ (7). In line with this interpretation another tribunal ruled that it was not reasonable for an investor to expect Lithuania, a country in the process of EU accession (so undergoing significant legislative change), to remain in a legislative freeze (7). Since tribunals are not obliged to follow a precedent when deciding on a dispute, different interpretations of the FET can be expected to continue (7). Thus when PMI also claimed violation of their right to FET arguing that it reasonably expected to be able to continue to use branding and trademarks on packaging to differentiate its products, it was difficult for Australia and other countries also considering plain packaging to predict the likely tribunal decision on this point (6).

**Most favoured nation**

The Most Favoured Nation (MFN) provision means investors are entitled to treatment as favourable as that provided to other investors covered under any investment treaty the government is party to (7). While this seems relatively straightforward, in recent decades MFN obligations have been used by investors to ‘import’ more favourable commitments from any of the other possibly dozens of agreements to which the host is party to replacing or supplementing the protections granted to the investor under the primary treaty (6, 7). Investors have successfully argued that although the IIA between their home country and the host may not be favourable to their claim, they are entitled to equally favourable treatment as promised to investors covered under other treaties the host country has signed (7). Some arbitral decisions also indicate that when a provision is ‘imported’, the investor can disassociate them from their original limitations and exceptions arguably allowing investors to generate a set of protections that enhances their rights to which the host country never agreed (7). The TPPA draft explicitly stated that parties are free to invoke provisions within other agreements/treaties that provide greater investor protection (10). This mean tobacco companies in one TPP country could still initiate an ISDS case against another using a more favourable agreement, effectively rendering the tobacco exception within the TPP ineffective (10).

**Unreasonable or discriminatory measures**

Usually a secondary claim to FET, ‘unreasonable or discriminatory measures’. In its claims against Australia, Phillip Morris attempted to argue that given the weak evidential link between plain packaging and the stated public health objectives, the measure was arbitrary or unreasonable and went on to claim any benefits from the legislation were disproportionate to the harm it would cause Phillip Morris’ investment (1). Similar claims were made in relation to Uruguay’s graphic warning label regulation (1). The Australian case was dismissed on jurisdictional grounds, but the tribunal in the Uruguay case drew heavily on the amicus brief submitted by the WHO and concluded that the rationale for the regulation was supported by public health evidence and was therefore not unreasonable or discriminatory (1).

**National treatment**

National Treatment (NT) is another core principle of international investment law and is designed to prohibit discrimination of foreign over local investors at all stages of the investment from the pre-establishment phase to post-establishment (1).

**References**

1. Mercurio B. Awakening the Sleeping Giant: Intellectual Property Rights in International Investment Agreements. Journal of International Economic Law. 2012;15(3):871-915. <https://doi.org/10.1093/jiel/jgs032>

2. International Centre for Settlement of Investment Disputes. Philip Morris Brands S´arl, Philip Morris Products S.A. and Abal Hermanos S.A. and Oriental Republic of Uruguay: award. ICSID case no. ARB/10/7; International Centre for Settlement of Investment Disputes. 2016. Available from: https://www.italaw.com/sites/default/files/case-documents/italaw7417.pdf

3. Hope J. Investor-state dispute settlment and tobacco control: implications for non-communicable diseases prevention and consumption-control measures. QUT Law Review. 2017;17(2):102-30. <https://doi.org/10.5204/qutlr.v17i2.709>.

4. Matveev A. Investor-state dispute settlement: The evolving balance between investor protection and state sovereignty. The University of Western Australia Law Review. 2015;40: 348- 386. Available from: https://www.law.uwa.edu.au/__data/assets/pdf_file/0006/2834736/13.-Arseni-Matveev-Investor-State-Dispute-Settlement-The-Evolving-Balance-between-Investor-Protection-and-State-Sovereignty.pdf

5. Sapiro M. Transatlantic trade and investment negotiations: Reaching a consensus on investor-state dispute settlement. Washington, DC: Brookings Institute; 2015. Available from: https://www.brookings.edu/research/transatlantic-trade-and-investment-negotiations-reaching-a-consensus-on-investor-state-dispute-settlement/

6. Stumberg R. Safeguards for tobacco control: options for the TPPA. Am J Law Med. 2013;39(2-3):382-441. https://doi.org/10.1177/009885881303900210.

7. Bernasconi-Osterwalder N, Cosbey A, Johnson L, VIs-Dunbar D. Investment treaties & Why they matter to sustaInable Development: Questions & Answers

. Winnipeg, Canada: International Institute for Sustainable Development; 2012. Available from: https://www.iisd.org/publications/investment-treaties-and-why-they-matter-sustainable-development-questions-and-answers

8. Fooks G, Gilmore AB. International trade law, plain packaging and tobacco industry political activity: the Trans-Pacific Partnership. Tobacco control. 2014;23(1):e1. https://doi.org/10.1136/tobaccocontrol-2012-050869

9. Mitchell A, Wurzberger SM. Boxed In? Australia's Plain Tobacco Packaging Initiative and International Investment Law. Arbitration International. 2011;27(4). https://doi.org/10.1093/arbitration/27.4.623

10. Labonté R, Schram A, Ruckert A. The Trans-Pacific Partnership Agreement and health: few gains, some losses, many risks Globalization and Health. 2016;12(25). https://doi.org/10.1186/s12992-016-0166-8

11. Weiss M. Trading Health? UK Faculty of Public Health Policy Report on the Transatlantic Trade and Investment Partnership. London: UK Faculty of Public Health; 2015. Available from: https://www.fph.org.uk/media/1380/fph-policy-report-on-the-transatlantic-trade-and-investment-report-final.pdf

12. Gaukrodger D. The balance between investor protection and the right to regulate in investment treaties: A scoping paper. Paris: OECD Publishing; 2017. Available from: https://www.oecd-ilibrary.org/deliver/82786801-en.pdf?itemId=%2Fcontent%2Fpaper%2F82786801-en&mimeType=pdf
